# Training of the Specialist

**Published:** 1913-10

**Authors:** Thomas J. Harris

**Affiliations:** New York


### Training of the Specialist.

Dr. Thomas J. Harris, New York: A concentrated advance movement to properly standardize our specialty is essential. The time is now ripe. As an outline for a more definite working basis, some such plan of instruction for special work may consist in the following divisions: First, thorough preparation in the undergraduate medical school; second, a general preliminary training through several years of practice or hospital interneship; third, at least six months of special instruction covering not only the clinical side, but the anatomic, pathologic, surgical, etc., as well; fourth, eighteen months of interneship in a special hospital or in the ear, nose and throat department of a general hospital; fifth, at the conclusion an examination by a university which should confer upon the successful candidate a postgraduate degree. The committee appointed to investigate and report on this subject is strongly of the opinion that the postgraduate instruction should be given in some established university, and further, that to make this departure a success, legislation will be necessary. The committee is of the opinion that any success in this movement must be the result of concerted action on the part of the several societies representing America and Canada.

